# Suicide in Massachusetts

**Published:** 1860-05

**Authors:** 


SUICIDE IN MASSACHUSETTS.—We append the subjoined statement from the *New York Observer*, of March 29th, 1860:—

"In the year 1858, 91 suicides occurred in Massachusetts, and the number has not greatly varied for the past five years. For the seventeen years and eight months ending Dec. 31, 1858, 1089 persons have taken their own lives in that State."

